# Left Atrial Appendage Closure vs. Oral Anticoagulation in Patients With Atrial Fibrillation: An Updated Systematic Review and Meta‐Analysis of Randomized Controlled Trials

**DOI:** 10.1002/joa3.70382

**Published:** 2026-06-19

**Authors:** Chika Chilaka, Hassan Dawood Khan, Muhammad Uzair Sarfraz, Zainab Farooq, Ebaad Hassan, Rabeea Sabir, Saman Siddique, Nimra Zahid, Mujeeb Ur Rehman, Muhammad Salih, Mohammad Umer, Asma'a Munasar Ali Alsubari, Muhammad Ehsan

**Affiliations:** ^1^ Lagos State University Nigeria; ^2^ Rashid Latif Medical College Lahore Pakistan; ^3^ King Edward Medical University Lahore Pakistan; ^4^ Punjab Rangers Teaching Hospital Lahore Pakistan; ^5^ Allama Iqbal Medical College Lahore Pakistan; ^6^ Jinnah Postgraduate Medical Center Karachi Pakistan; ^7^ National Hospital and Medical Center Lahore Pakistan; ^8^ Khyber Teaching Hospital Peshawar Pakistan; ^9^ Dow University Hospital Karachi Pakistan; ^10^ Faculty of Medicine Sana'a University Sana'a Yemen

**Keywords:** atrial fibrillation, direct oral anticoagulants, DOAC, LAAC, left atrial appendage closure, left atrial appendage occlusion, vitamin K antagonist, warfarin

## Abstract

**Background:**

While oral anticoagulants (OACs) remain the cornerstone of stroke prevention, left atrial appendage closure (LAAC) is a potential alternative, particularly for patients with high bleeding risk or contraindications to long‐term anticoagulation.

**Methods:**

Multiple databases were searched to identify relevant randomized controlled trials (RCTs), and four trials were shortlisted. Study selection, data extraction, and quality assessment were performed independently by two reviewers. Outcomes assessed included stroke (all, ischemic, hemorrhagic), systemic embolism, major bleeding, non‐procedure‐related bleeding, and mortality. Risk of bias was evaluated using Rob 2.0, and meta‐analyses were conducted using RevMan software. Dichotomous outcomes were reported as risk ratios (RR) while continuous outcomes were reported as mean differences (MD), with 95% confidence intervals (CIs).

**Results:**

A total of 3116 participants were analyzed across outcomes. LAAC significantly reduced non‐procedure‐related bleeding (RR: 0.48, 95% CI: 0.37–0.61; *p* < 0.00001) and all‐cause mortality (RR: 0.74, 95% CI: 0.55–0.99; *p* = 0.04). A marginal reduction was observed in the composite outcome of stroke, systemic embolism, or death (RR: 0.77, 95% CI: 0.59–1.00; *p* = 0.05). No significant differences were found for major bleeding (RR: 0.82, 95% CI: 0.56–1.22), all strokes (RR: 0.84, 95% CI: 0.56–1.25), ischemic stroke (RR: 1.15, 95% CI: 0.72–1.85), hemorrhagic stroke (RR: 0.46, 95% CI: 0.11–2.02), systemic embolism (RR: 1.52, 95% CI: 0.36–6.41), or cardiovascular/unexplained death (RR: 0.60, 95% CI: 0.29–1.23).

**Conclusions:**

LAAC offers a viable alternative to standard anticoagulation in selected AF patients with comparable efficacy and a particular benefit in reducing non–procedure‐related bleeding and mortality advantages.

## Graphical abstract

Efficacy and safety outcomes from meta‐analysis of left atrial appendage closure versus anticoagulation therapy in atrial fibrillation. Pooled analysis of 3116 participants from four randomized trials demonstrated comparable composite outcome of stroke, systemic embolism, and cardiovascular/unexplained mortality between LAAC and standard anticoagulation (RR 0.84, *p* = 0.05), with superior bleeding reduction (RR 0.48, *p* < 0.00001) and mortality advantage (RR 0.74, *p* = 0.04). LAAC represents a viable alternative for high‐bleeding‐risk patients.

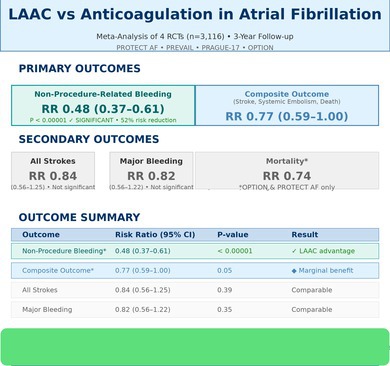

## 1 Introduction

Atrial fibrillation (AF) is one of the most common arrhythmias seen in adults and its incidence is rising exponentially [1, 2, 3, 4]. Around 3–6 million people are estimated to have AF in the United States and this number is expected to rise to 6–16 million by the year 2050 [4, 5]. Several different risk factors can result in AF, with age being the most important non‐modifiable risk factor [6]. Hypertension, diabetes, and lifestyle factors are also known to play a major role in the pathophysiology of AF [6]. Patients can be asymptomatic or may present with dizziness, palpitations, diaphoresis, chest pain, or shortness of breath. The most feared complications of AF are stroke, embolism, and heart failure [7].

Treatment of AF is based on two main approaches: rate control and rhythm control [8]. To prevent the complications of AF, anticoagulants like vitamin K antagonists (VKAs) and non‐VKA oral anticoagulant (NOAC) are used [9]. Direct oral anticoagulants (DOACs) are preferred over vitamin K antagonists in most patients due to improved safety profiles, particularly lower rates of intracranial hemorrhage.

Despite their proven efficacy, long‐term anticoagulation carries a clinically significant bleeding risk and remains contraindicated or poorly tolerated in a subset of patients [10]. Furthermore, real‐world adherence to OAC therapy is suboptimal, and bleeding concerns frequently lead to treatment discontinuation. As the majority of thrombi in non‐valvular AF originate from the left atrial appendage, device‐based strategies targeting this structure have emerged as alternative approaches to stroke prevention [11].

Left atrial appendage closure (LAAC), including percutaneous techniques such as implantation of the WATCHMAN device, has been evaluated in multiple randomized controlled trials comparing its efficacy and safety to long‐term anticoagulation [12, 13, 14, 15]. Long‐term follow‐up data from landmark trials have demonstrated non‐inferiority to warfarin for prevention of stroke and systemic embolism, with potential reductions in hemorrhagic stroke and major non‐procedural bleeding. More recent trials have expanded comparisons to direct oral anticoagulants and higher‐risk populations, suggesting evolving clinical indications beyond patients with absolute contraindications to OAC [16, 17].

However, several important questions remain unresolved. Individual trials vary in patient selection, comparator anticoagulant strategy (warfarin vs. DOAC), duration of follow‐up, and definitions of clinical endpoints. Moreover, contemporary practice has shifted toward DOAC‐dominant anticoagulation, necessitating reassessment of LAAC efficacy relative to current standard‐of‐care therapy rather than historical comparators alone. While some analyses suggest reductions in hemorrhagic stroke and long‐term mortality with LAAC, uncertainty persists regarding ischemic stroke risk, procedural complications, and overall net clinical benefit in broader AF populations.

Given these evolving data, an updated and comprehensive meta‐analysis of randomized controlled trials is warranted. By pooling contemporary RCT evidence comparing LAAC with guideline‐directed oral anticoagulation, this study aims to provide a higher level of evidence regarding efficacy, safety, and overall clinical outcomes. Specifically, we sought to evaluate stroke and systemic embolism, non‐procedural major bleeding, all‐cause and cardiovascular mortality, device‐related complications, and net clinical benefit to clarify the role of LAAC in modern AF management and to better inform shared decision‐making in patients at varying thromboembolic and bleeding risks.

## 2 Materials and Methods

This systematic review and meta‐analysis was conducted in accordance with the Preferred Reporting Items for Systematic Reviews and Meta‐Analyses (PRISMA) guidelines and the Cochrane Handbook for Systematic Reviews of Interventions. The study protocol was prospectively registered on PROSPERO (ID: 1128470).

### 2.1 Eligibility Criteria

We included RCTs that compared LAAC with standard care (anticoagulation therapy) in patients with AF, and reported relevant clinical outcomes. Studies were excluded if they were observational in nature, literature reviews, meta‐analyses, editorials, case reports, or animal studies. No restrictions were applied regarding language or geographic location.

### 2.2 Information Sources and Search Strategy

A comprehensive literature search was performed across multiple electronic databases, including MEDLINE (Pubmed), Embase, and ClinicalTrials.gov, from inception through June 2025. We used a combination of keywords and MeSH terms such as “left atrial appendage closure,” *“LAAC,” “atrial fibrillation,” “anticoagulation,” “DOAC,”* and “randomized controlled trials.” Boolean operators AND and OR were applied to refine the search. We also manually screened reference lists of relevant articles and systematic reviews to identify additional eligible studies.

### 2.3 Study Selection

Search results were uploaded to Rayyan, a web‐based platform for systematic reviews. After removing duplicates, two independent reviewers screened all titles and abstracts. Full texts of potentially eligible studies were then reviewed based on the inclusion criteria. Discrepancies were resolved through discussion, with a third reviewer consulted when necessary.

### 2.4 Data Extraction

Selected studies were imported into Zotero (version 6.0.30). Two reviewers independently extracted data using a pre‐tested data extraction sheet, and a third reviewer verified the entries. Extracted data included participant demographics, study characteristics (design, location, year), intervention and comparator details, and outcome measures.

### 2.5 Risk of Bias Assessment

The revised Cochrane Risk of Bias tool for RCTs (RoB 2.0) was used to assess the quality of included studies. Two reviewers independently evaluated the risk of bias across five domains: Randomization process, deviations from intended interventions, missing outcome data, outcome measurement, selection of reported results. Disagreements were resolved through discussion followed by consensus.

### 2.6 Endpoints

The primary outcomes assessed in this study included non‐procedure‐related bleeding (trial defined); a composite of stroke, systemic embolism, or death from cardiovascular, unexplained, or all‐cause mortality. The composite outcomes reported by the included trials differed slightly with some reporting stroke and systemic embolism in combination with cardiovascular/unexplained death, and others in combination with all‐cause mortality. Moreover, one trial reported bleeding along with the above‐mentioned outcomes. All of the trials reported each component of their composite outcomes separately as well. Consequently, we had to use the component outcomes to add data to our pre‐defined composite outcome where the trial's reported composite outcome did not fully align with ours. Additional outcomes included major bleeding events (trial‐defined); all strokes; ischemic stroke; hemorrhagic stroke; systemic embolism; the number and percentage of individuals with device‐related adverse events; device embolization; device‐related vascular complications; device‐related death; and device‐ or procedure‐related pericardial effusion. Device‐related outcomes were presented as frequency and percentage since they were invariably reported in the LAAC arms. We also analyzed deaths attributed specifically to cardiovascular or unexplained causes, as well as overall all‐cause mortality.

### 2.7 Synthesis of Data

Meta‐analyses were conducted using Review Manager (RevMan) version 5.4. A random‐effects model with the DerSimonian‐Laird estimator was employed. Statistical significance was set at *p* ≤ 0.05. Risk ratios (RRs) were used for dichotomous outcomes and mean differences (MDs) for continuous data, both reported with 95% confidence intervals (CIs). Heterogeneity was evaluated using the I^2^ and Chi^2^ statistic, interpreted per Cochrane recommendations. We planned to assess publication bias using the funnel plot if the number of studies were more than 10 in any of the analyses. For the primary outcomes, subgroup analysis was conducted based on agent used for anticoagulation (warfarin vs. DOACs), CHA_2_DS_2_VASc score (≥ 4 vs. < 4) and HAS‐BLED score (≥ 2 vs. < 2). Sensitivity analysis was conducted by excluding the study conducted on post‐ablation AF patients to see if the reduced (presumed) AF burden in these patients would meaningfully change the results.

## 3 Results

### 3.1 Study and Participant Characteristics

**FIGURE 1:**
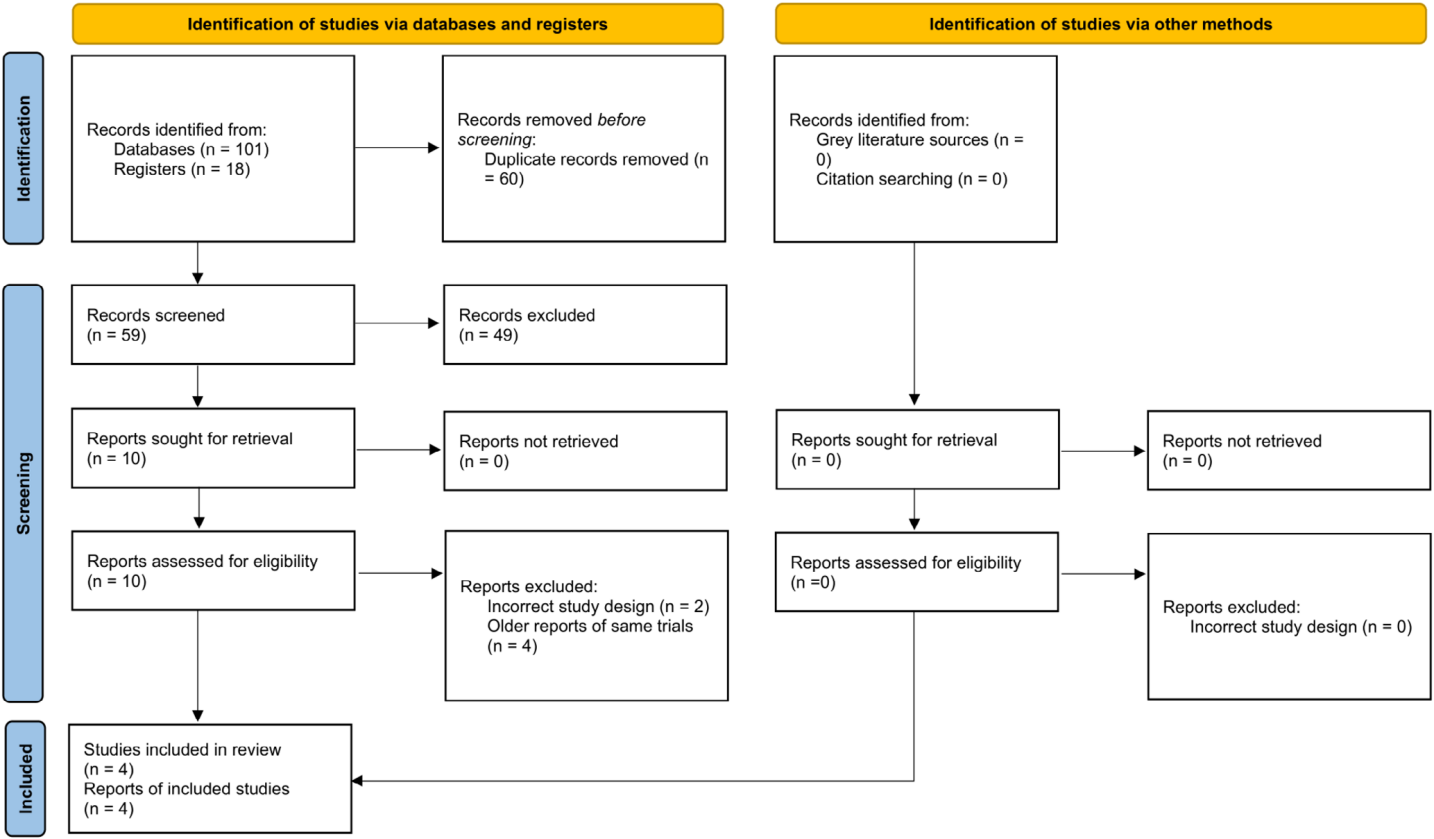
PRISMA flowchart for study selection.

A total of 119 studies were found after searching through various databases. Four RCTs were shortlisted and included in this meta‐analysis after full text‐screening was completed. These studies were selected based on an inclusion criterion made prior to the search strategy. The selection process is illustrated using a PRISMA flowchart (Figure 1).

The OPTION trial [12] included 1600 patients, 803 of whom were randomized into the LAAC group and 797 of whom were put in the anticoagulant (control) treatment group. This was the only trial in this meta‐analysis that solely enrolled participants who had undergone catheter ablation for atrial fibrillation. The intervention group had 520 males, and the total mean age was 69.7 years, whereas the control group had 533 males with a total mean age of 69.4 years. There were 707 patients in the PROTECT AF trial [13], which was a multicenter, open‐label, non‐inferiority study. 463 participants were randomized to the LAAC group and 244 into the warfarin control group. The average age of participants in the LAAC group was 71.7 years (70.4% male), whereas the average age of participants in the control group was 72.7 years (70.1% male). The PREVAIL trial [14], which was a follow‐up trial to PROTECT AF with improved procedural safety, had 407 patients, 269 of whom underwent LAAC, and 138 of whom were administered warfarin. The average age of participants in the LAAC group was 74.0 years (67.7% male), whereas the average age of participants in the control group was 74.9 years (74.6% male). The PRAGUE study [15] had 402 patients, with 201 in the LAAC group and 201 in the DOAC group. The mean age was 73.4 years in the LAAC group with 134 males and 73.2 years in the DOAC group (130 males). This study included participants with an overall higher bleeding risk compared to the rest of the included studies. Refer to Table 1 for detail.

**TABLE 1:** Study and participants' characteristics.

Study ID, origin, design	Duration	Sample size	Mean age (mean ± SD)	Males (%)	CHADS ≥ 3 or CHADSVASc ≥ 2	CHADSVASC mean score	HASBLED mean score	AF pattern	Intervention	Comparator
OPTION Multicenter Open label design	36 months	803 vs. 797	69.7 ± 7.4 vs. 69.4 ± 7.9	520 (66.8%)‐533 (66.9%)	828 vs. 790	3.5 ± 1.3 vs. 3.5 ± 1.3	1.2 ± 8 vs. 1.2 ± 8	Persistent 326 vs. 296 Paroxysmal 477 vs. 501	Left Atrial Appendage closure using WATCHMAN	Oral Anticoagulation at physician's discretion
PRAGUE Multicenter Open label design	30 months	201 vs. 181	73.2 ± 7.2 vs. 73.4 ± 6.7	130 (64.7%) 134 (66.7)	201 vs. 201	4.7 ± 1.5 vs. 4.7 ± 1.5	3.0 ± 0.9 vs. 3.1 ± 0.9	Paroxysmal 67 vs. 53 persistent 46 vs. 47 long standing persistent 16 vs. 18 permanent 72 vs. 83	Left atrial appendage closure using WATCHMAN or Amulet	Direct Oral anticoagulants
PROTECT AF Multicenter Open label design	18 months	463 vs. 244	72.7 ± 9.2 years	70%	149 vs. 90	NA	NA	Paroxysmal 43% vs. Persistent 21% vs. Permanent 35%	Left atrial appendage closure (LAAC) using WATCHMAN.	Warfarin
PREVAIL Multicenter Open label design	5 years	269 vs. 138	74.0 ± 7.4 vs. 74.9 ± 7.2	67.7 vs. 74.6	269 vs. 138	3.8 ± 1 vs. 3.9 ± 1	NA	Paroxysmal 131 vs. 71 Permanent 42 vs. 22 Persistent 85 vs. 39 Unknown 4 vs. 1	Left atrial appendage closure using WATCHMAN	Warfarin

Abbreviations: AF, Atrial fibrillation; NA, Not applicable.

**FIGURE 2:**
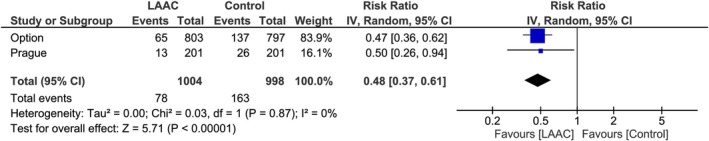
Forest plot depicting effect estimate for non‐procedure‐related bleeding.

**FIGURE 3:**
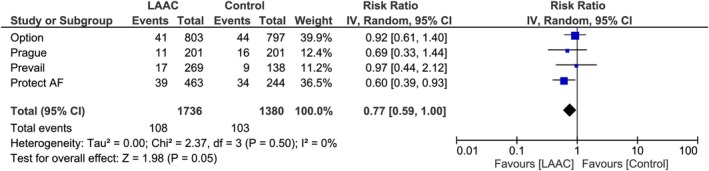
Forest plot depicting effect estimate for composite outcome.

**FIGURE 4:**
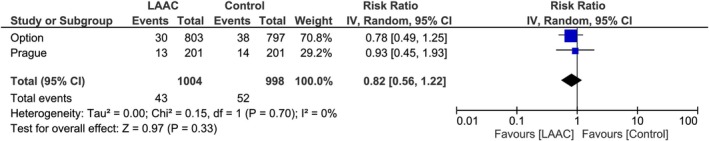
Forest plot depicting effect estimate for major bleeding.

**FIGURE 5:**
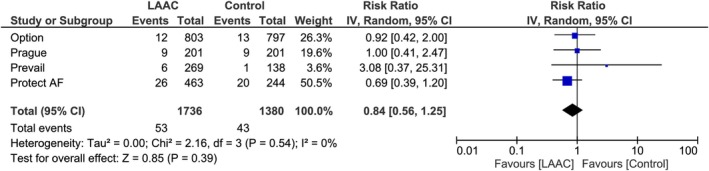
Forest plot depicting effect estimate for all strokes.

### 3.2 Risk of Bias Assessment

All the RCTs had a low risk of bias in randomization, selection, and reporting. However, because of the way the intervention was set up, the studies were inherently unblinded, which in our estimation did not introduce a significant bias.

### 3.3 Results of Meta‐Analysis

LAAC was associated with a significantly lower risk of non–procedure‐related bleeding compared to the control group (RR 0.48, 95% CI 0.37–0.61; *p* < 0.00001; I^2^ = 0%; Figure 2). These results remained consistent across subgroups compared on the basis of anticoagulant agent (test for subgroup differences *p* = 0.97), CHA_2_DS_2_VASc score (*p* = 0.49), and HAS‐BLED score (*p* = 0.48). Sensitivity analysis conducted by excluding the OPTION study did not alter the results (*p* < 0.00001). Although a 23% relative risk reduction was observed for the composite endpoint of stroke, systemic embolism, or death from cardiovascular or unexplained causes, this did not reach statistical significance, with the upper bound of the confidence interval approaching 1 (RR 0.77, 95% CI 0.59–1.00; *p* = 0.50; I^2^ = 0%; Figure 3), suggesting the need for further evidence to validate this trend. These results remained consistent in subgroup analysis based on anticoagulant agent used (*p* = 0.87), CHA_2_DS_2_VASc score (*p* = 0.83), and HAS‐BLED score (*p* = 0.68). Sensitivity analysis conducted by excluding the OPTION study pushed the results to statistical significance (*p* = 0.02).

The incidence of major bleeding was also not significantly different between groups. While the point estimate favored LAAC with an 18% relative risk reduction, the wide confidence interval (RR 0.82, 95% CI 0.56–1.22; *p* = 0.70; I^2^ = 0%; Figure 4) indicated that the true effect could range from a 44% reduction to a 22% increase.

Both all‐stroke (RR 0.84, 95% CI 0.56–1.25; *p* = 0.54; I^2^ = 0%; Figure 5) and ischemic stroke (RR 1.15, 95% CI 0.72–1.85; *p* = 0.82; I^2^ = 0%) rates did not differ significantly between groups. Hemorrhagic stroke demonstrated a trend favoring LAAC (RR 0.46, 95% CI 0.11–2.02; *p* = 0.14; I^2^ = 49%), though the wide confidence interval precluded definitive conclusions. Similarly, systemic embolism occurred more frequently in the LAAC group (RR 1.52, 95% CI 0.36–6.41; *p* = 0.74; I^2^ = 0%), but the estimate was imprecise and likely reflected random variation.

Mortality outcomes generally trended in favor of LAAC. Cardiovascular or unexplained death showed a nonsignificant reduction (RR 0.60, 95% CI 0.29–1.23; *p* = 0.07; I^2^ = 70%), whereas all‐cause mortality was significantly reduced with LAAC (RR 0.74, 95% CI 0.55–0.99; *p* = 0.05; I^2^ = 0%).

Device‐related events had very low event rates or limited reporting. Among device‐related adverse events, incidence rates were low: device‐related complications occurred in 0.23% of participants, device embolization and device‐related vascular complications each occurred in 0.50%, device‐related mortality in 0.20%, and pericardial effusion linked to the device or procedure in 0.40% of participants.

## 4 Discussion

### 4.1 Summary of Main Findings

This meta‐analysis evaluated the efficacy and safety of Left Atrial Appendage Closure (LAAC) compared to standard anticoagulation therapy for stroke prevention in atrial fibrillation. The most robust finding was a significant reduction in non‐procedure‐related bleeding with LAAC (RR: 0.48, 95% CI: 0.37 to 0.61, *p* < 0.00001), observed with high consistency (I^2^ = 0%) across 1004 LAAC participants and 998 controls from the OPTION and PRAGUE‐17 trials. This indicates LAAC approximately halves the risk of bleeding events not tied to implantation, offering a notable benefit for patients at high bleeding risk or with poor adherence to oral anticoagulants (OACs). For the composite endpoint of stroke, systemic embolism, or cardiovascular/unexplained/all‐cause mortality, a borderline significant reduction was found with LAAC, with no heterogeneity (I^2^ = 0%) across 1736 LAAC recipients and 1380 controls. No significant differences were observed for overall major bleeding, all strokes, ischemic strokes, hemorrhagic stroke, systemic embolism, or cardiovascular/unexplained death. These findings suggest LAAC is a viable alternative to anticoagulation, particularly for reducing long‐term bleeding, with potential broader benefits for mortality and composite outcomes, relevant to clinicians, patients, and policymakers managing atrial fibrillation. This interpretation holds even when LAAC is compared to different anticoagulation agents including warfarin, an anti‐coagulant that is believed to have a worse safety index when compared to DOACs. Moreover, the results of our subgroup analysis demonstrate the non‐inferiority of LAAC among patients with a higher CHA_2_DS_2_VASc and HAS‐BLED score.

### 4.2 Interpretation of Findings in the Context of Other Evidence

The significant reduction in non‐procedure‐related bleeding aligns with LAAC's mechanistic advantage of eliminating long‐term systemic anticoagulation, a primary driver of bleeding in atrial fibrillation patients. Landmark trials like PROTECT AF and PREVAIL, which compared LAAC to warfarin, demonstrated reduced major bleeding (excluding peri‐procedural events) over extended follow‐up [18]. Real‐world data from the NCDR LAAO Registry further confirm this benefit, reporting low peri‐procedural bleeding rates (below 2%) with devices like the Watchman FLX, even when Direct Oral Anticoagulants (DOACs) are part of standard care [19]. Similarly, the EWOLUTION Registry, evaluating the Watchman device, supports sustained bleeding reductions in high‐risk patients [20]. The 2023 ACC/AHA/ACCP/HRS Guideline for the Diagnosis and Management of Atrial Fibrillation has formally incorporated this evidence, recommending LAAC (Class 2a) for patients with a long‐term contraindication to OAC and (Class 2b) as a potential alternative for patients with a high risk of bleeding who are able to tolerate a short course of post‐procedural anticoagulation [21].

The borderline significant reduction in the composite endpoint of stroke, systemic embolism, or mortality is consistent with PROTECT AF's long‐term non‐inferiority to warfarin and suggests broader benefits [13, 18]. Recent studies, including PRAGUE‐17 and OPTION, affirm LAAC's non‐inferiority to DOACs for ischemic stroke and systemic embolism [12, 15]. In a recent meta‐analysis, Jiang et al. observed significantly lower rates of all‐cause (HR 0.68) and cardiovascular mortality (HR 0.55) with LAAC compared to DOACs [22]. While the difference in stroke or transient ischemic attack events did not reach statistical significance, the trend favored LAAC, suggesting a potential benefit that warrants further exploration. However, Elsheikh S et al. found similar 5‐year risks for mortality, ischemic stroke, and intracranial hemorrhage in propensity‐matched LAAC and DOAC cohorts, highlighting variability in outcomes based on study design [23]. The current meta‐analysis is an update on both of these studies by including data from the OPTION trial which included 1600 participants, thus increasing the sample size and the power of this analysis. Two new trials (CHAMPION‐AF and CLOSURE‐AF) published after study selection was completed for this analysis. The CHAMPION‐AF study demonstrated non‐inferiority of LAAC compared to DOACs in a composite outcome of cardiovascular death, stroke, and systemic embolism (1.20 95% CI 0.87 to 1.66) for Atrial fibrillation in patients who were candidates for oral anticoagulation. These conclusions bring forth the idea of LAAC as a primary treatment option instead of an alternative for these patients. The CLOSURE‐AF trial recruited patients with a much higher bleeding and coagulation risk compared to the populations studied thus far. The study did not demonstrate non‐inferiority of LAAC to physician‐directed best medical care (85% received DOACs) in a composite outcome of cardiovascular/unexplained death, stroke, systemic embolism *and* major bleeding. Secondary outcomes separately comparing the components of the composite primary outcome did not demonstrate a significant statistical difference. These results may contribute to a more patient‐dependent approach to the choice between LAAC and oral anticoagulation in patients with higher clotting risks. This aligns perfectly with the 2023 ACC/AHA/ACCP/HRS guideline's cautious stance, which acknowledges emerging evidence of potential mortality benefits but refrains from making a recommendation based on this endpoint due to outcome variability. The guideline appropriately prioritizes LAAC for its established safety profile in high‐bleeding‐risk patients over uncertain mortality gains [21].

For major bleeding, the lack of significant difference (RR: 0.82) reflects a balance between low peri‐procedural bleeding risks, as seen in the Amulet IDE trial (approximately 1%–2% with contemporary devices) [24, 25], and long‐term bleeding reductions. Registries like NCDR LAAO and EWOLUTION report declining peri‐procedural complications due to improved technology and operator experience [19, 20], supporting the potential for LAAC's long‐term benefits to outweigh initial risks. The Amulet IDE trial further demonstrates low complication rates with devices like the Amplatzer Amulet [24]. The 2023 ACC/AHA/ACCP/HRS guideline explicitly discusses this balance, noting that procedural risks are minimized in experienced centers, thereby making LAAC a viable long‐term strategy for patients in whom the persistent threat of OAC‐related bleeding is a primary concern [21].

Regarding stroke subtypes, the non‐significant findings for all strokes and ischemic strokes align with LAAC's established non‐inferiority to OACs, including DOACs, for ischemic stroke prevention [18]. The numerical increase in ischemic stroke risk (RR: 1.15) noted in some studies may be linked to incomplete LAA seal or device‐related thrombus (DRT), as reported by Fauchier et al. and Boersma et al. [20, 26]. The 2023 ACC/AHA/ACCP/HRS guideline addresses this by recommending rigorous post‐procedural imaging. The guideline also highlights hemorrhagic stroke reduction as a key advantage of LAAC in patients with elevated bleeding risk [21]. While our study observed a non‐significant reduction in hemorrhagic stroke (RR: 0.46), this trend aligns with larger meta‐analyses, including that by Oliva et al. [27], which found significant reductions, reinforcing the potential role of LAAC in carefully selected high‐risk populations.

The result for all‐cause mortality trending in favor of LAAC needs cautious interpretation given that the effect was borderline and the number of events was limited. Moreover, the trials reporting this outcome separately were not adequately powered for it and it is unlikely that this pooled analysis has compensated for it. However, it still remains interesting, and future more‐adequately powered trials may help create a clearer picture.

The heterogeneity in cardiovascular/unexplained death (I^2^ = 70%) likely stems from variations in patient characteristics (e.g., CHA_2_DS_2_‐VASc, HAS‐BLED scores), comparator types (warfarin vs. DOACs), endpoint definitions, device types, and follow‐up duration [23].

### 4.3 Limitations of This Meta‐Analysis

Several limitations impact the interpretation of this meta‐analysis. At the study level, substantial heterogeneity (I^2^ = 70%) for cardiovascular/unexplained death complicates unified conclusions, potentially driven by differences in patient demographics, control treatments, endpoint definitions, or follow‐up duration. Low event rates for outcomes like hemorrhagic stroke and systemic embolism likely limited statistical power, reducing the ability to detect significant differences. Variable follow‐up durations across studies may have missed long‐term outcome divergences, particularly for major bleeding and the composite endpoint. The mix of control treatments (warfarin in older studies vs. DOACs in newer ones) could have influenced pooled estimates; however, the subgroup analyses did not demonstrate a significant effect. Additionally, one of the included trials (OPTION) studied post‐ablation participants only, while another trial (PRAGUE‐17) included participants with an overall higher bleeding risk compared to the other included studies. These factors could have had an unpredictable effect on the outcomes of this study, although our sensitivity analysis did not attest to that. Finally, the inherent design of the included trials made blinding impossible, which could have introduced a bias in results reporting for patient‐reported outcomes.

At the review level, our search was limited to English‐language publications, potentially introducing language bias. We did not formally assess publication bias due to the small number of studies for some outcomes, which may have affected results. The use of aggregate data precluded detailed subgroup analyses, limiting insights into specific patient populations. These limitations highlight the need for cautious interpretation and further research to address these gaps.

### 4.4 Implications for Clinical Practice and Future Research

The 2023 ACC/AHA/ACCP/HRS guidelines are in line with our findings. LAAC is a reasonable alternative for stroke prevention in atrial fibrillation patients with high bleeding risk or contraindications to long‐term OAC. Clinicians should apply a shared decision‐making framework, incorporating CHA_2_DS_2_‐VASc and HAS‐BLED scores to guide patient selection. While emerging data suggest improved composite outcomes, these findings remain exploratory. Careful procedural technique and adherence to post‐implantation protocols are essential to minimize risks such as device‐related thrombus [20, 26].

Future research should prioritize long‐term, adequately powered studies comparing LAAC with contemporary DOAC therapy—such as the ongoing CATALYST trial (NCT04226547), which evaluates the Amplatzer Amulet device in patients without contraindications to anticoagulation. Additionally, extended follow‐up from real‐world registries like EWOLUTION will help clarify the safety and efficacy of modern LAAC devices in diverse populations. Targeted studies focusing on high‐risk subgroups (e.g., prior stroke, elevated bleeding risk) and strategies to mitigate ischemic stroke risk post‐LAAC—such as optimized device selection, procedural technique, and post‐implant antithrombotic regimens—are essential to refine patient selection and improve outcomes.

## 5 Conclusion

This meta‐analysis demonstrates that LAAC significantly reduces non‐procedure related bleeding compared to standard anticoagulation, reinforcing its role in stroke prevention for high‐risk atrial fibrillation patients. Although superiority for composite ischemic and mortality outcomes was not established, LAAC's non‐inferior efficacy and potential mortality benefit are consistent with recent findings [18, 23]. Ongoing trials and registries—such as CATALYST and EWOLUTION—are expected to provide further insight into the long‐term safety and efficacies of LAAC, while continued refinement of patient selection and procedural strategies remains essential.

## Author Contributions

Chika Chilaka: data, curation, investigation, methodology, formal analysis, resources, writing – original draft preparation. Hassan Dawood Khan: data curation, formal analysis, methodology, writing – original draft preparation. Muhammad Uzair Sarfraz: methodology, data curation, writing – original draft preparation. Zainab Farooq: methodology, resources, writing – original draft preparation. Ebaad Hassan: Formal analysis, Investigation, writing – original draft preparation. Rabeea Sabir: visualization, data curation, writing – reviewing and editing. Saman Siddique: methodology, resources, writing – original draft preparation. Nimra Zahid: investigation, resources, writing – original draft preparation. Mujeeb Ur Rehman: Methodology, data curation, writing – original draft preparation. Muhammad Salih: resources, data curation, writing –.review and editing. Mohammad Umer: conceptualization, project administration, visualization, supervision, writing – review and editing. Muhammad Ehsan: conceptualization, supervision, writing – review and editing.

## Funding

The authors have nothing to report.

## Disclosure

Human and Animal Participants: Research involving human participants and/or animals: No animals or human subjects were used in the current study.

## Consent

The authors have nothing to report.

## Conflicts of Interest

The authors declare no conflicts of interest.

## Data Availability

The data that support the findings of this study are available from the corresponding author upon reasonable request.
